# Quality Characteristics and Flavor Analysis of Five Mulberry Varieties

**DOI:** 10.3390/foods13244088

**Published:** 2024-12-17

**Authors:** Yingmei Meng, Yinyin Lian, Jiaxin Li, Huayi Suo, Jiajia Song, Mei Wang, Yu Zhang

**Affiliations:** 1 School of Food Science, Southwest University, Chongqing 400700, China; mengyingmei140@sina.com (Y.M.); lianyinyin@email.swu.edu.cn (Y.L.); swulijx@email.swu.edu.cn (J.L.); birget@swu.edu.cn (H.S.); songjiajia1208@126.com (J.S.); 2 National Teaching Demonstration Center of Food Science and Engineering, Southwest University, Chongqing 400700, China; 3 Sericultural Science and Technology Research Institute, Chongqing 400700, China; ximeiga@163.com; 4 National Citrus Engineering Research Center, Southwest University, Chongqing 400712, China

**Keywords:** mulberry variety, total phenol, total flavonoid, volatile components

## Abstract

For a deeper understanding of the characteristics exhibited by several novel mulberry varieties, the quality attributes and flavor components of five mulberry varieties (Zhongsang 5801, 2000-3, Jialing 40, Yuesang 10, and White Shahtoot Mulberry) were analyzed and compared. Zhongsang 5801 displayed the highest total phenol and flavone levels. Fructose and glucose were the primary sugars identified in the mulberries, with 2000-3 exhibiting the highest fructose content (39.66 ± 11.31 g/kg), whereas Zhongsang 5801 had the highest glucose content (26.19 ± 6.29 g/kg). The key organic acids found in the five mulberry varieties were oxalic acid, tartaric acid, and malic acid. Of them, 2000-3 had the highest malic acid content (0.66 ± 0.02 g/kg). Furthermore, 21 amino acids and 66 volatile components were detected in the five mulberry varieties. The study findings offer valuable insights for assessing, processing, and utilizing different mulberry varieties.

## 1. Introduction

Mulberry (family: Moraceae) is a deciduous tree plant producing sweet and delicious fruits with a unique flavor [1]. The fruits have a high nutritional value and abundant vitamins, minerals, and other beneficial nutrients [2]. They contain various components such as flavonoids, anthocyanins, carotenes, and polysaccharides that exhibit diverse human health-promoting biological activities [3]. All parts of the mulberry tree, including leaves, root bark, branches, and fruits, are used in traditional Chinese medicine practices, which maximizes their utilization value [4]. Moreover, numerous studies have reported the anti-inflammatory and antioxidant effects of mulberries [5], obesity [6] and diabetes [7] combating potential, neuroprotective properties [8], atherosclerosis-preventing ability [9,10], blood pressure-lowering effects [11], and liver-protecting capabilities [12], among other notable attributes. Because of its pleasant taste profile combined with unique flavors and exceptional nutritional content, the demand for mulberries in medicinal applications and dietary consumption has been increasing [13].

Mulberry is extensively cultivated globally, and various mulberry varieties have adapted to different ecological and climatic regions, resulting in abundant resources [14]. On a global scale, the cultivation techniques and management methods for mulberries are diversified according to different regions, climates, and soil conditions. In Asia, especially China, India, and Southeast Asia, mulberry has a long history of cultivation, and has accumulated rich planting experience and a unique variety resources [15]. In Europe and the Americas, although the cultivation of mulberries started late, thanks to advanced agricultural technology and strict quality management, the quality and flavor of mulberries have been continuously improved [16,17].

With the development of globalization and trade, the international trade volume of mulberries is also increasing year by year. Mulberry varieties, quality, and flavor in different countries and regions have their own characteristics, providing consumers with a variety of choices. However, this also puts forward higher requirements for the quality and flavor analysis of mulberries. In the field of mulberry quality and flavor analysis, researchers have conducted in-depth research on many aspects of mulberry through modern scientific and technological means. For example, Di Ma used SBSE-GC/Q-TOF-MS techniques to identify and quantify volatiles in 14 mulberry cultivars [18]. Ali Ali Redha analyzed the mineral content of mulberry trees using inductively coupled plasma emission spectroscopy (ICP-OES) to reveal the nutritional value and chemical composition of mulberry varieties [19]. Junwei Huo used liquid chromatography high resolution time-of-flight mass spectrometry (LC-HRTOF/MS2) to identify and quantify 11 anthocyanins and 20 non-anthocyanin phenolic compounds, and conducted a comprehensive analysis of the structure and bioactivity of “Heisang No. 1” polyphenols [20]. These studies not only revealed the material basis of the quality and flavor of mulberry, but also provided ascientific basis for the breeding, cultivation, and processing of mulberry.

Various mulberry cultivars are available worldwide. However, only limited information has been reported regarding their quality characteristics [21]. The quality characteristics of five mulberry varieties with a high local yield, namely, White Shahtoot Mulberry, Zhongsang 5801, 2000-3, Jialing 40, and Yuesang 10, have not been investigated in detail. In addition to being eaten as fruit, mulberries can also be used to make fruit juice, fruit wine, jam, and other foods. By studying mulberry varieties, we can understand the characteristics and advantages of different varieties in processing and utilization, and provide a scientific basis for the diversified utilization of mulberry. Therefore, this study analyzed the physical and chemical properties, nutritional components, and flavor profiles of the aforementioned five mulberry varieties. The study offers a reference for evaluating the quality of select mulberry varieties and facilitating the development and utilization of related products while promoting further exploration and exploitation of mulberry resources.

## 2. Materials and Methods

### 2.1. Mulberry Sample Preparation

**Figure 1:**
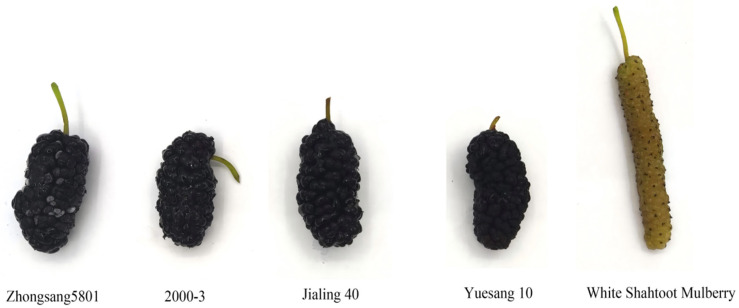
Image of five mulberry varieties.

The experimental materials were White Shahtoot Mulberry, Zhongsang 5801, 2000-3, Jialing 40, and Yuesang 10 (Figure 1). White Shahtoot Mulberry was selected from the Chongqing Sericulture Science and Technology Research Institute (latitude: 29°50′38.328″ N, longitude: 106°26′45.60″ E). Zhongsang 5801, 2000-3, Jialing 40, and Yuesang 10 were selected from the Yongchuan District of Chongqing (latitude: 29°21′21.960″ N, longitude: 105°55′37.218″ E). The planting distance of mulberry trees in the two plantations was 1.67 m and the row distance was 1.33 m. All five kinds of mulberry trees were 15 years old. Mulberry was collected at 2 PM on 13 April 2024. Multiple sampling points were randomly selected in the mulberry planting area, 500 g of mulberries were randomly collected at each sampling point, and the collected mulberries were evenly mixed to form experimental materials. A total of 5 kg of mulberries were collected. Some fresh mulberries were used for the determination of total acids, total phenols, total flavonoids, and volatile substances immediately after picking, while others were frozen and stored for the determination of other indexes.

### 2.2. Determination of Physicochemical Properties

Titratable acidity was measured through titration with 0.01 mol/L NaOH and expressed as citric acid equivalents. The end point of titration was reached at a pH of 8.2. The 50 g of mulberries were ground with 50 g of distilled water to make a homogenate. A total of 25 g homogenate were weighed, 50 mL distilled water were added, the sample was boiled in a water bath for 30 min and cooled down, and distilled water was added to bring the liquid volume up to 250 mL. The filtered solution was used to determine titratable acids. The pH level was measured using a pH meter (PHS-3E, Shanghai INESA Scientific Instrument Co., Ltd., Shanghai, China). The soluble solid content was quantified using a handheld refractometer (WZS-32, Shanghai YIDen Physical Optics Instrument Co., Ltd., Shanghai, China). Each treatment was repeated 3 times.

The total sugar content was determined through anthrone colorimetry [22]. In this procedure, 5 g of mulberry were ground, and 25 mL of distilled water were added to a triangular flask and extracted through ultrasonication for 10 min. The resulting mixture was filtered. The filtrate was collected in a 100 mL volumetric flask and subsequently filled up to the calibration line. After the sample solution was diluted by a factor of 100, 1 mL of the diluted solution was mixed with 4 mL of a sulfuric acid reagent containing anthrone (2.0 g/L; prepared with 80% sulfuric acid) and placed in a boiling water bath for exactly 10 min. Finally, the absorbance of the mixture was measured at 620 nm by using a visible spectrophotometer (Shanghai Jinghua Science and Technology Instrument Co., Ltd., Shanghai, China). A glucose standard solution (purity ≥ 98%, Shanghai Shifeng Biotechnology Co., Ltd., Shanghai, China) was used to plot the calibration curve. The result was calculated according to the standard curve equation. Each treatment was repeated 3 times.

### 2.3. Nutrional Index Measurements

#### 2.3.1. Determination of Total Phenol Content

The total phenol content was measured using the Folin phenol reagent method with modifications [23]. First, 5 g of mulberry were accurately weighed and ground, and transferred to a 50 mL volumetric bottle, and then anhydrous ethanol was added to the scale. Sample solution was obtained by filtration after ultrasonic treatment for 10 min. One mL of sample solution, 6 mL of deionized water, and 1 mL of a 1 mol/L Folin phenol reagent were combined in each 25 mL graduated test tube and shaken for 6 min. The mixture was then allowed to stand. Subsequently, 4 mL of 10.6% sodium carbonate solution was added to the test tube, shaken, and incubated at room temperature for 60 min. Finally, the volume was adjusted with deionized water to attain the desired scale. The absorbance of each sample was measured at 760 nm by using a visible spectrophotometer (Shanghai Jinghua Science and Technology Instrument Co., Ltd., Shanghai, China). The gallic acid standard solution (purity ≥ 98%, Shanghai Shifeng Biotechnology Co., Ltd., Shanghai, China) was used for calibration. The result was calculated according to the standard curve equation. Each treatment was repeated 3 times.

#### 2.3.2. Determination of Total Flavonoid Content

The total flavonoid content was measured using the aluminum nitrate method as described previously [24]. First, mulberries were ground, diluted by 5 times with 80% ethanol water, subjected to ultrasonic treatment for 30 min, and filtered to form a diluent. One mL of the diluted sample was added to a 25 mL colorimetric tube, along with 5 mL of 30% ethanol and 1 mL of 5% sodium nitrite solution. The mixture was vigorously shaken, reacted for 6 min, and supplemented with 1 mL of 10% aluminum nitrate solution. The mixture was shaken again and incubated for 6 min. The precisely measured quantities (10 mL) of 1 mol/L sodium hydroxide solution were then added to this mixture. Finally, the colorimetric tube was filled up to the desired level with additional amounts of the aforementioned 30% ethanol and left undisturbed for 15 min. A blank control without samples was used as a reference for the absorbance analysis of the reaction mixture at 510 nm by using a visible spectrophotometer (Shanghai Jinghua Science and Technology Instrument Co., Ltd., Shanghai, China). The rutin standard solution (purity ≥ 98%, Fuzhou Feijing Biological Technology Co., Ltd., Fuzhou, China) was used for calibration. The result was calculated according to the standard curve equation. Each treatment was repeated 3 times.

### 2.4. Flavor Component Analysis

#### 2.4.1. Determination of Soluble Sugar

Analysis of soluble sugars was performed by high-performance liquid chromatography (HPLC) with an Athena NH2-RP column (250 mm × 4.6 mm, 5 µm) and a refractive index detector after slightly modifying Gundogdu et al.’s method (2011). The 10 g of mulberries were accurately weighed and then transferred to a 100 mL volumetric bottle after grinding. About 50 mL of distilled water were added, ultrasound was performed for 5 min, and then centrifugation (8000× *g*, 10 min) and filtering was carried out. All the filtrate was collected in a 100 mL volumetric bottle, and distilled water was added to bring the liquid volume up to 100 mL. The sample was filtered with a 0.22 μm filter membrane. The mobile phase was a 7:3 ratio of acetonitrile to water (volume ratio), the column temperature was 40 °C, the flow rate was 1.0 mL/min, and the sample size was 10 µL. Standards of fructose, glucose, sucrose, maltose, and lactose (fructose, glucose, sucrose, maltose, and lactose, purity ≥ 98%, Shanghai Shifeng Biotechnology Co., Ltd., Shanghai, China) were used to make standard curves, and the measured results corresponded to the standard curves, so as to calculate the concentration. Each treatment was repeated 3 times.

#### 2.4.2. Determination of Organic Acid

Organic acids were determined using a method modified based on the original HPLC method [25]. The sample was prepared by adding an equal volume of deionized water to a weighed 5 g homogenate, followed by the addition of 15 mL of 80% ethanol solution. The mixture was then incubated in a water bath at 75 °C for 0.5 h, cooled to room temperature, and transferred to a 25 mL volumetric flask with deionized water. The mixture was centrifuged at 9000× *g* for 30 min, and the resulting supernatant was filtered through a pinhead filter membrane (pore size: 0.22 µm). Chromatographic separation was performed using a C18 column (250 mm × 4.6 mm, particle size: 5 µm, Agilent, Santa Clara, CA, USA) with a mobile phase comprising a solution containing sodium dihydrogen phosphate (pH = 2.8) and methanol in a 97:3 ratio (*v*/*v*). A flow rate of 1 mL/min was maintained throughout the analysis while approximately 20 µL of sample were injected into the column inlet system. The concentration of organic acids was determined by measuring their UV absorbance at 210 nm. Different concentrations of oxalic acid, tartaric acid, malic acid, lactic acid, ascorbic acid, acetic acid, citric acid, and succinic acid (oxalic acid, tartaric acid, malic acid, lactic acid, ascorbic acid, acetic acid, citric acid, and succinic acid, purity ≥ 98%, Shanghai Shifeng Biotechnology Co., Ltd., Shanghai, China) were used to make standard curves, and the measured results corresponded to the standard curves so as to calculate the concentration. Each treatment was repeated 3 times.

#### 2.4.3. Determination of Amino Acid

The amino acid content was assessed through HPLC by using a C18 column (250 mm × 4.6 mm, 5 µm, Agilent, USA) with UV detection equipped [26]. The 20 g sample was dried at 80 °C, crushed, dissolved with 0.1 mol/L hydrochloric acid, ultrasonicated for 40 min, and then filled with 0.1 mol/L hydrochloric acid in a 25 mL volumetric bottle. After filtration, 2 mL of supernatant were extracted and 2.5 mL of amino acid mixed standard solution were obtained, and 0.625 mL of derivative A (phenyl isothiocyanate 2 mL, acetonitrile 8 mL) and 2.5 mL of derivative B (triethylamine 2 mL, acetonitrile 4 mL) were added, respectively. After reaction at room temperature and in a dark place for 1 h, 5 mL of n-hexane were added for extraction twice, for 10 min each time. The lower solution was absorbed and mixed with 100 μL of acetic acid solution and filtered by a 0.22 μm filter membrane. The chromatographic conditions used were mobile phase A consisting of 0.1 mol/L sodium acetate solution:acetonitrile (97:3, *v*/*v*) (acetic acid adjusted pH was 6.5) and mobile phase B consisting of acetonitrile:water (4:1, *v*/*v*), with a flow rate of 1 mL/min and a gradient elution of 0–11 min, 0–1.5% A, 100–98.5% B; 11–21.7 min, 1.5–7.6% A, 98.5–92.4% B; 21.7–23.9 min, 7.6–11% A, 92.4–89% B; 23.9–39 min, 11–30% A, 89–70% B; 39–42 min, 30–70% A, 70–30% B; 42–45 min, 70–100% A, 30–0% B; 45–52 min, 100% A, 0% B; 52–55 min, 100–0% A, 0–100% B; and 55–70 min, 0% A, 100% B. The detection wavelength was 254 nm, the column temperature was 36 °C, and the sample size was 20 µL. The standard solution of 21 kinds of amino acids (purity ≥ 98%, Shanghai Amp Golden Standard Technical Service Co., Ltd., Shanghai, China) was taken as the standard curve. The amino acid concentration of the sample was calculated by aligning the measured value with the standard curve. Each treatment was repeated 3 times.

#### 2.4.4. Extraction and Determination of Volatile Components

The concentration of volatile components in mulberries was evaluated using the modified version of previous procedures [27]. The volatile components were analyzed through solid-phase microextraction. Briefly, 5 g of mulberry samples were placed in a 20 mL headspace bottle, supplemented with 1.5 g of NaCl, and incubated at 55 °C for 10 min. Subsequently, the samples were treated with a solution containing 60 µL of 2-octanol and bathed at 55 °C for 20 min. A SPME probe was positioned in a headspace vial at approximately 20 mm above the sample surface to adsorb the volatiles within 20 min. Immediately after the extraction, the loaded SPME fiber (50/30 µm DVB/CAR/PDMS Headspace Solid Phase Microextraction fiber head, Supelco, Bellefonte, PA, USA) was removed from the sample and injected into a gas chromatography–mass spectrometry instrument (GCMS-QP2010 gas chromatography–mass spectrometry instrument, Shimadzu, Kyoto, Japan).

The volatiles were separated using a DB-5MS column (30 m × 0.25 mm × 0.25 mm). Initially, the oven temperature was set at 40 °C for 5 min and increased to 120 °C at a rate of 4 °C/min. The temperature then ramped up from 120 °C to 210 °C at a rate of 6 °C/min (held for 9 min at 210 °C), and finally increased to 240 °C at a rate of 25 °C/min (held for an additional 3 min). The total runtime was approximately 55 min. Highly pure helium gas was used as the carrier gas, with a flow rate of 1.0 mL/min. The ion source temperature was maintained at 250 °C, and the ionization voltage was set to 70 eV with a 35 *m/z*-to-450 m/z scanning range. The GC-MS experimental data were qualitatively obtained through similarity retrieval in the NIST18.0 database. The data were semi-quantified by using 2-octanol (purity ≥ 99.5%, Shanghai Maclin Biochemical Technology Co., Ltd., Shanghai, China) as an internal standard, and the concentration of each compound was calculated as follows: component content = internal standard concentration × peak area of each component/peak area of an internal standard. Each treatment was repeated 3 times.

### 2.5. Statistical Analysis

All measurements were conducted in triplicate and expressed as mean ± standard deviation (SPSS statistics 27.0). Analysis of variance with Duncan’s multiple comparison was performed to assess the significance of sample effects. The difference was considered significant when *p* < 0.05. Origin 2018 (OriginLab, Northampton, MA, USA) and ChiPlot (https://www.chiplot.online/, (accessed on 21 August 2024)) were used to generate all the graphs in the manuscript.

## 3. Results and Discussion

### 3.1. Titratable Acidity, Soluble Solid, and Total Sugar

**Figure 2:**
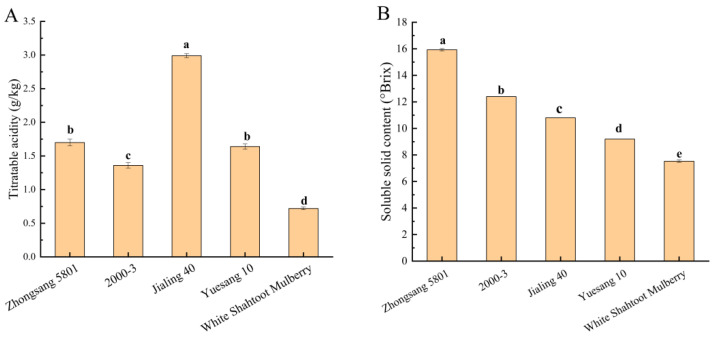

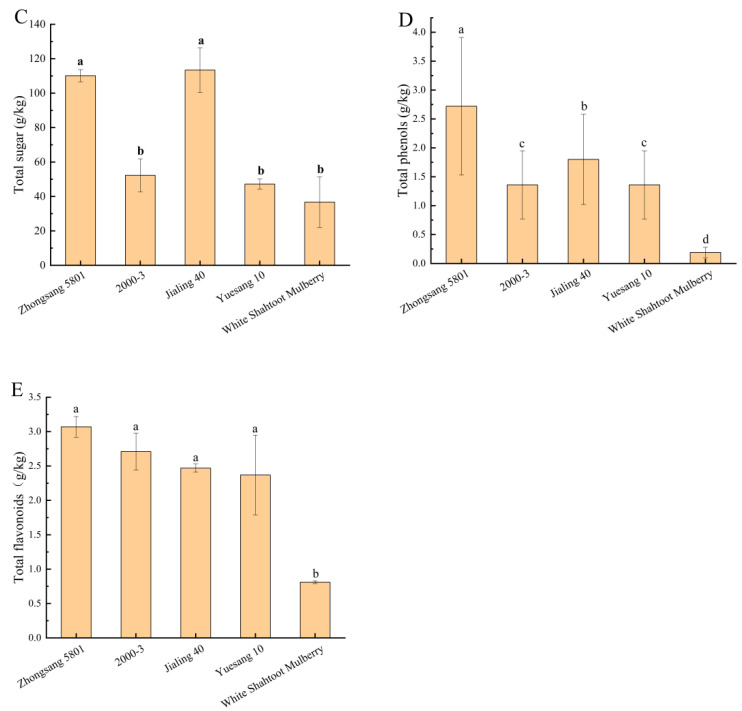
Physical and chemical indices and nutritional indices of five mulberry varieties. (**A**) Titratable acidity (g/kg). (**B**) Soluble solid content (°Brix). (**C**) Total sugar content. (**D**) Total phenol content (g/kg). (**E**) Total flavonoid content (g/kg). Different lowercase letters indicate significant differences among varieties (*p* < 0.05).

Titratable acidity, soluble solid content, and total sugar content are the key factors attributable to fruit taste. A knowledge of these characteristics of diverse mulberry varieties can help producers select specific raw mulberry materials to improve product quality and consumer acceptance. The titratable acidity was significantly higher in Jialing 40 than in the other four mulberry varieties (*p* < 0.05) (Figure 2A). The soluble solid content is another crucial factor determining mulberry quality. It includes soluble sugars and other soluble components. The soluble solid content of the five mulberry varieties differed significantly, with Zhongsang 5801 having the highest content (15.93 ± 0.09), and °Brix and White Shahtoot Mulberry having the lowest content (7.53 ± 0.09) (Figure 2B). The total sugar content was significantly higher in Zhongsang 5801 and Jialing 40 than in the other three varieties (*p* < 0.05) (Figure 2C). The total sugar content of Zhongsang 5801 and Jialing 40 was approximately twice that of the other three mulberry varieties. The total sugar content measured in this experiment ranged from 36.7 to 113.41 g/kg, which is similar to the content (129.88 ± 1.09 g/kg) of mature “Dashi” mulberry measured in previous experiments [22]. In general, mulberries with lower acidity and higher soluble solid and total sugar contents taste sweeter. Thus, Zhongsang 5801 is seemingly more popular among consumers who prefer a sweeter taste of mulberries.

### 3.2. Total Phenols and Total Flavonoids

The total phenol and flavonoid contents are functional components of mulberry. These factors have pivotal effects on human nutrition and health [28,29]. The total phenol content of different mulberry varieties varied. The total phenol content was significantly higher in Zhongsang 5801 (2.72 ± 1.19 g/kg) (Figure 2D) than in the other four varieties (*p* < 0.05). In other studies, the total phenol content ranged from 3.23 to 5.30 g/kg for different mulberry varieties [30]. In our study, the total phenol content of several mulberries was determined. The total phenol content ranged from 0.19 to 2.72 g/kg (Figure 2D), which is relatively lower than those noted in the other varieties. This difference may be attributable to genetic variations and environmental conditions [31]. The total flavonoid content was found to descend in order of magnitude in the five varieties as follows: Zhongsang 5801, 2000-3, Jialing 40, Yuesang 10, and White Shahtoot Mulberry. The total flavonoid content ranged from 0.81 to 3.07 g/kg (Figure 2E). According to Bao et al.’s study, the total flavonoid content of both commonly cultivated and newly discovered mulberry varieties in China ranged from 0.37 to 2.6 g/kg fresh weight, consistent with our findings [21]. Among these varieties, Zhongsang 5801 exhibited the highest total and flavonoid contents, which indicates the potential of this mulberry variety as a valuable resource for developing functional beverages and foods.

### 3.3. Soluble Sugar and Organic Acid

As an energy source, soluble sugar is a major fruit component and an intrinsic quality of fruits [32]. Glucose and fructose were the key sugars in the five mulberry varieties examined. The fructose content detected in all mulberries was greater than the glucose content, indicating that fructose contributes more to sweetness in these mulberries (Table 1). Lou et al. found that the fructose content of three mature mulberry fruits accounted for the highest proportion. Our findings are consistent with theirs [33]. Although the fructose content of Zhongsang 5801 was not the highest, it was 38.89 ± 6.55 g/kg (Table 1). The difference, however, was not significant compared with the variety with the highest fructose content (*p* > 0.05). The glucose content of Zhongsang 5801 was the highest (26.19 ± 6.29 g/kg) (Table 1), and no significant difference was observed among the varieties with the second and third highest glucose contents (*p* > 0.05). The fructose content of five kinds of mulberries was measured to be in the range of 19.75–39.66 g/kg. There was a partial overlap with the 12.66–62.77 g/kg fructose content measured by Lou et al. in five mulberry fruit varieties suitable for cultivation in tropical areas, which indicates the reliability of the results of soluble sugar in this experiment [33].

Organic acids play a pivotal role in forming fruit flavor [34]. Oxalic acid, tartaric acid, and malic acid were detected in all mulberry varieties, which indicated significant variations among the five mulberry varieties. Among these organic acids, the relative content of malic acid was the highest, and it served as the primary organic acid in mulberries. Malic acid is an indispensable intermediate of the tricarboxylic acid (TCA) cycle in plants and significantly influences the quality of fruit flavors [35]. The malic acid content was significantly higher in 2000-3 than in other mulberry varieties, measuring 1.85 ± 0.05 g/kg (Table 1, *p* < 0.05), followed by White Shahtoot Mulberry, Yuesang 10, and Zhongsang 5801. By contrast, the lowest concentration was observed in Jialing 40 (1.26 ± 0.07 g/kg) (Table 1). These findings suggest variations in the metabolic pathways of the TCA cycle among these five mulberry varieties, which leads to inconsistent accumulation of intermediates [36]. Citric acid, tartaric acid, succinic acid, lactic acid, fumaric acid, and acetic acid were detected in white mulberry, black mulberry, and red mulberry, among which the malic acid content was 13.23–44.67 g/kg [37]. The content of malic acid measured in this experiment was 1.26–1.85 g/kg, which is lower than the previously reported value. Furthermore, only malic acid, oxalic acid, tartaric acid, and acetic acid were detected. The organic acid content among species may vary because of genetic factors, cultivation practices, and environmental conditions such as temperature, light intensity, and humidity [38,39].

### 3.4. Amino Acid

Amino acids play a major role as essential nutrients and flavor enhancers. The quantity and arrangement of amino acids are substantial determinants of food taste [40]. In total, 21 amino acids were identified in mulberries. The amino acid measurements closely align with those reported by Jiang Yan et al. (2015) (Table 2). White Shahtoot Mulberry had the highest total amino acid content (5689.05 ± 134.6 mg/kg), followed by Zhongsang 5801, Jialing 40, Yuesang 10, and 2000-3 in descending order of magnitude. Of these varieties, essential and non-essential amino acids were present in the highest quantities in White Shahtoot Mulberry (908.01 ± 43.7 mg/kg and 4382.07 ± 86.63 mg/kg, respectively). The 2000-3 variety had the lowest quantities of essential and non-essential amino acids (229.69 ± 14.73 mg/kg and 2147.01 ± 398.82 mg/kg, respectively).

Significant variations were observed in the free amino acid profiles of the five mulberry varieties. The highest theanine, glycine, alanine, and proline contents were observed in Zhongsang 5801. The highest glycine and serine contents were found in 2000-3. The highest glutamine and isoleucine contents were found in Jialing 40. The highest aspartic acid and valine contents were observed in Yusang 10. The highest glutamic acid, asparagine, threonine, histidine, arginine, methionine, leucine, aminobutyric acid, and phenylalanine contents were observed in White Shahtoot Mulberry. Notably, White Shahtoot Mulberry had significantly higher concentrations of glutamic acid (811.48 ± 40.87 mg/kg), asparagine (1406.01 ± 87.42 mg/kg), and aminobutyric acid (368.86 ± 5.81 mg/kg) than the other varieties (*p* < 0.05). Glutamic acid contributes to the umami taste perception in fruits. Among the studied varieties, Zhongsang 5801 had the highest proline content at 88.06 ± 6.09 mg/kg, which was approximately seven times that of Yuesang 10. Jialing 40 had significantly higher glutamine levels, at 265.65 ± 54.93 mg/kg, than the other varieties tested herein. A higher alcohol content is the by-product of alcoholic beverage fermentation, and its high content is harmful to physiological health. Lian et al. found that a higher content of alcohol was significantly negatively correlated with the content of corresponding amino acids [41]. The content of amino acids of White Shahtoot Mulberry was higher than that of the other four mulberry varieties. White Shahtoot Mulberry may be a better choice as the raw material of fermented mulberry wine.

**Figure 3:**
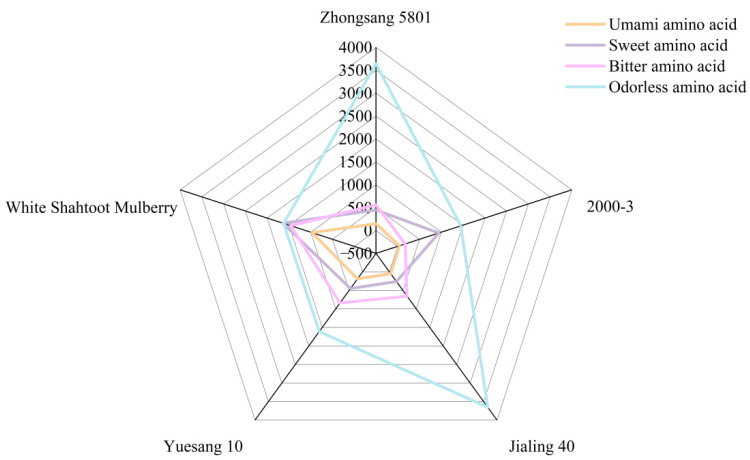
Radar image of amino acid taste characteristics of different mulberry varieties. The digital scale on the gray line on the radar map indicates the concentration of amino acids in mg/kg.

Based on the taste characteristics of amino acids, mulberry tastes can be categorized into four groups: umami, sweet, bitter, and odorless [42,43]. The content of flavorless amino acids in the four classification groups was the highest in all mulberries except White Shahtoot Mulberry (Figure 3). The sweet amino acid content was the highest in White Shahtoot Mulberry, followed by 2000-3. Sweet amino acids can enhance the sweetness of food while also contributing to umami promotion [44].

### 3.5. Volatile Components

**Figure 4:**
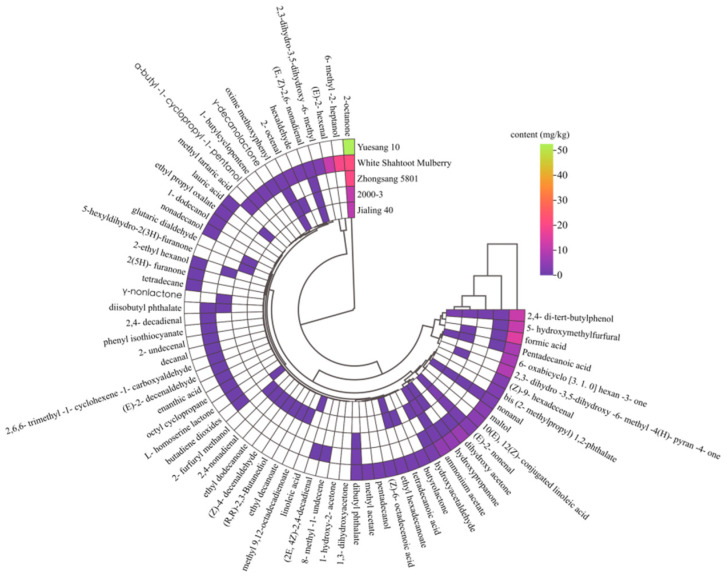
Hierarchical cluster analysis of main volatile components. The color in the figure represents the content of volatile substances. The more purple the color, the lower the content, and the more green the color, the higher the content.

Volatile components play a pivotal role in fruit flavor. These components usually originate from the chemical components of the fruit itself, such as esters, alcohols, and aldehydes. They thus give a unique flavor and taste to the fruits [45]. Overall, 66 types of volatile components were detected in all mulberry varieties, including esters (14), alcohols (8), aldehydes (18), ketones (7), acids (9), and other volatile components (10). The types and contents of volatile components in different mulberry varieties were quite varied (Figure 4). In total, 11, 25, 12, 31, and 36 volatile components were detected in Zhongsang 5801, 2000-3, Jialing 40, Yuesang 10, and White Shahtoot Mulberry, respectively.

**Figure 5:**
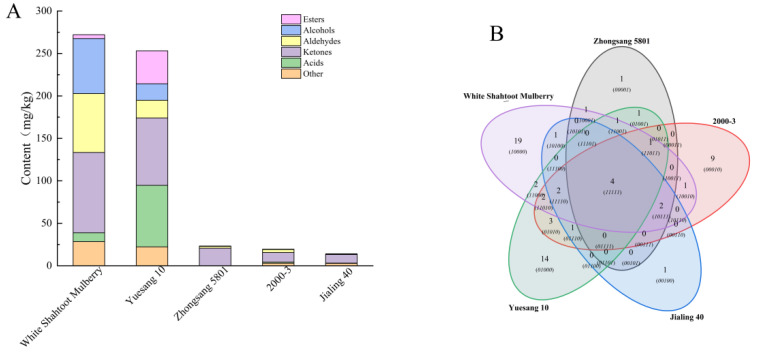
Contents and types of volatile components in the five mulberry varieties. (**A**) Classification and content of volatile compounds in different mulberry varieties. (**B**) Venn diagram of volatile substances.

The distribution of different volatile components in each mulberry variety is presented in Figure 5A. Among all varieties, Yuesang 10 had the highest content of total volatile components, followed by White Shahtoot Mulberry, Zhongsang 5801, 2000-3, and Jialing 40. In Figure 5B, variations in volatile components among the different mulberry varieties can be clearly observed. The unique components of White Shahtoot Mulberry, Yuesang 10, 2000-3, Jialing 40, and Zhongsang 5801 were 19, 14, 9, 1, and 1, respectively. The OAV value of each volatile flavor substance was calculated by the ratio of concentration to odor threshold. A total of 22 volatile flavor substances with OAV > 1 were identified. There were 5, 9, 4, 6, and 13 kinds of Zhongsang 5801, 2000-3, Jialing 40, Yuesang 10 and White Shahtoot Mulberry, respectively (Table 3). Generally speaking, OAV > 1 is considered to be the characteristic flavor substance, which has a greater contribution to the overall flavor [46].

Volatile ester components are crucial for shaping the aromatic profile of fruits. Some esters bring special flavors to fruits, such as γ-nonlactone, which has a buttery sweet taste, and ethyl caprate, which confers a fruity and apple-like odor. Thus, they contribute to the fundamental aroma characteristics of mulberries [65]. Among the 14 esters detected, 4 esters had an OAV > 1. They are γ-decanolactone, γ-nonlactone, butyrolactone, and ethyl decanoate. Gamma-decanolactone provides White Shahtoot Mulberry with fat, fruit, lactone, and peach flavors. The unique gamma-nonlactone of Zhongsang 5801 provides apricot and fruit flavors. Butyrolactone can provide caramel, cheese, and roasted nut flavors to Yuesang 10. The unique Ethyl decanoate of 2000-03 provides brandy, grape, and pear flavors.

Alcohol is the main flavor component that contributes to the light flavor of mulberry fruits. The total content of alcohol components was the highest in White Shahtoot Mulberry, followed by Yuesang 10, 2000-3, Zhongsang 5801, and Jialing 40 (Figure 5A). Among alcohols, only 6-methyl-2-heptanol and 1-dodecanol have an OAV > 1, but their flavor description is unclear. 6-Methyl-2-heptanol was abundant in White Shahtoot Mulberry, at 19.83 ± 8.63 mg/kg (Supplementary Table S1). 

Aldehydes significantly contribute to the complexity of fruit flavors [66]. In total, 20 aldehydes were identified in the five varieties, including nonanal and (E)-2-nonenal. Hexaldehvde (0–1.58 mg/kg), 2-octenal (0–0.11 mg/kg), (E)-2-nonenal (0.51–3.07 mg/kg), 2,4-nonadienal (0–0.12 mg/kg), and other compounds were relatively abundant in the five species of mulberries, which is consistent with previous studies [18]. Aldehydes such as hexaldehyde, nonanal, decanal, (E)-2-hexenal, (E)-2-nonenal, and (E)-2-octenal may be closely related to green odors, whereas general aldehydes containing 6–10 carbon atoms are related to both green and fatty aromas [67]. There are 12 aldehydes with an OAV > 1. Hexaldehyde, decanal, (E)-2-decenaldehyde, and 2,4-decadienal substances, unique to White Shahtoot Mulberry, provide it with apple, fat, fresh, orange peel, penetrating, coriander, and other flavors. The unique 2-Octenal, (Z)-4-decenaldehyde, and 2,4-nonadienal of 2000-3 provide dandelion, fat, fruit, grass, green, spice, cereal, and other flavors.

Ketones are volatile components, and a high total ketone content was detected in many mulberry varieties. Among the five varieties, Yuesang 10 had the highest ketone content (Figure 5A). Furthermore, ketones can give fruits a special smell, such as 5-hexyldihydro-2(3H)-furanone providing fruits a “buttery” and “sweet” flavor [18]. 2-Octanone can bring fat, fragrant, and mold flavors to mulberries.

The content of volatile acid components in Yuesang 10 was the highest (Figure 5A). Pentadecanoic acid was identified in 2000-3, Jialing 40, Yuesang 10, and White Shahtoot Mulberry. Among these mulberry varieties, Yuesang 10 contained the highest content of pentadecanoic acid, at 7.36 ± 2.91 mg/kg (Supplementary Table S1) in the acidic fraction. Despite this, pentadecanoic acid had an OAV < 1. This shows that although its content was relatively high, it had no significant contribution to the flavor of the mulberries.

In general, the 66 volatile components identified through GC-MS differed significantly in the distinct mulberry varieties. The high ester and aldehyde concentration distinguished Yuesang 10, whereas White Shahtoot Mulberry had higher alcohol concentrations than the other varieties. In this study, the quantities and contents of volatile components were higher in Yuesang 10 and White Shahtoot Mulberry than in the other three varieties. This suggests that Yuesang 10 and White Shahtoot Mulberry have richer and more intense aromas than the other three varieties. It is reported that fatty acids and amino acids are the precursors in the metabolic pathway of volatile components, and they can be converted into aromatic volatile substances through a series of biochemical reactions in the plant [68,69]. At present, there are few studies on their transformation pathway in mulberries, and it is still unclear. Future research could further explore the specific relationship between them. This provides some ideas for future research.

### 3.6. Correlation Analysis Between Amino Acids and Volatile Substances (OAV > 1)

**Figure 6:**
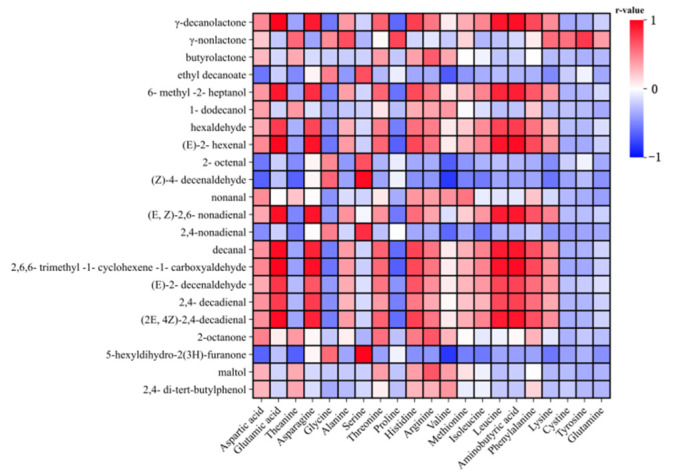
Pearson analysis between amino acids and volatile substances.

Pearson correlation analysis of 21 key volatile flavor compounds with 22 amino acids is shown in the heat map (Figure 6). In the figure, red and blue indicate positive correlation and negative correlation, respectively, and the depth of color indicates the level of correlation.

Glutamic acid, theanine, alanine, methionine, isoleucine, leucine, aminobutyric acid, phenylalanine, lysine, γ-decanolactone, 6-methyl-2-heptanol, hexaldehyde, (E)-2-hexenal, (E,Z)-2,6-nonadienal, decanal, 2,6,6-trimethyl-1-cyclohexene-1-carboxyaldehyde, (E)-2-decenaldehyde, 2,4-decadienal, and (2E,4Z)-2,4-decadienal showed a strong positive correlation. Aspartic acid, histidine, arginine, γ-decanolactone, butyrolactone, 6-methyl-2-heptanol, 1-dodecanol, hexaldehyde, (E)-2-hexenal, nonanal, (E,Z)-2,6-nonadienal, decanal, 2,6,6-trimethyl-1-cyclohexene-1-carboxyaldehyde, (E)-2-decenaldehyde, 2,4-decadienal, (2E,4Z)-2,4-decadienal, 2-octanone, maltol, and 2,4-di-tert-butylphenol were positively correlated. Proline, lysine, cystine, tyrosine, and glutamine showed strong negative correlation with other volatile substances except gamma-nonlactone. This suggests that the more amino acids that are negatively correlated with volatile substances, the more detrimental it is to the formation of these flavor compounds.

## 4. Conclusions

In this study, the physical and chemical components, and the total phenols, flavonoids, soluble sugars, organic acids, free amino acids, and volatile components of five mulberry varieties were investigated. The results demonstrate variations in the physical and chemical properties, nutritional components, and flavor profiles of the different mulberry varieties. Zhongsang 5801 exhibited superior characteristics, with higher soluble solid and soluble sugar contents than the other four varieties. Zhongsang 5801 also had the highest total phenol and total flavone concentrations, along with a greater abundance of active components. Glucose and fructose were the primary sugars identified in these mulberries. Zhongsang 5801 had higher glucose and fructose contents. Oxalic acid, tartaric acid, and malic acid were the predominant organic acids in all mulberry varieties investigated. Notably, 2000-3 had significantly higher malic acid levels than the other varieties. White Shahtoot Mulberry had both the highest concentration of total amino acids and the second highest concentration of volatile components, which contributed to its strong fragrance profile among all varieties. This study offers valuable insights into understanding the chemical compositions and flavor profiles of mulberries. These findings can serve as a foundation for raw material selection, quality studies, and the development and advancement of related products.

Soluble sugars, organic acids, amino acids, and volatile substances in mulberry fruits play an important role in nutritional value and flavor characteristics. Although there is currently little research on the specific connection between them, it can be speculated that there may be some interaction between them. Future research could further explore the specific relationship between them and how to improve the quality and flavor of mulberries by regulating their content and variety.

## Figures and Tables

**Table 1:** Soluble sugar content and organic acid content of different mulberries (g/kg).

	Cultivar	Zhongsang 5801	2000-3	Jialing 40	Yuesang 10	White Shahtoot Mulberry
Index						
Soluble sugar	Fructose	38.89 ± 6.55 ^a^	39.66 ± 11.31 ^a^	22.51 ± 2.90 ^ab^	19.75 ± 1.04 ^b^	31.19 ± 7.82 ^a^
	Glucose	26.19 ± 6.29 ^a^	18.63 ± 3.72 ^a^	6.43 ± 4.59 ^b^	16.25 ± 2.20 ^ab^	16.83 ± 4.44 ^a^
	Sucrose	-	-	-	-	-
	Maltose	-	-	-	-	-
	Lactose	-	-	-	-	-
Organic acid	Oxalic acid	0.07 ± 0.00 ^c^	0.10 ± 0.01 ^a^	0.08 ± 0.00 ^b^	0.07 ± 0.00 ^c^	0.03 ± 0.00 ^d^
	Tartaric acid	0.11 ± 0.01 ^b^	0.08 ± 0.00 ^c^	0.08 ± 0.00 ^c^	0.09 ± 0.00 ^bc^	0.16 ± 0.02 ^a^
	Malic acid	1.33 ± 0.01 ^d^	1.85 ± 0.05 ^a^	1.26 ± 0.07 ^d^	1.44 ± 0.01 ^c^	1.54 ± 0.07 ^b^
	Lactic acid	-	-	-	-	-
	Ascorbic acid	-	-	-	-	-
	Acetic acid	-	0.04 ± 0.01 ^a^	-	0.02 ± 0.00 ^a^	-
	Citric acid	-	-	-	-	-
	Succinic acid	-	-	-	-	-

“-” indicates that the device was not detected. The values of different lowercase letters in the same line differ significantly (*p* < 0.05).

**Table 2:** Amino acid content of different mulberries (mg/kg).

	Cultivar	Zhongsang 5801	2000-3	Jialing 40	Yuesang 10	White Shahtoot Mulberry
Index						
Umami amino acid	Aspartic acid	126.93 ± 10.80 ^b^	31.58 ± 13.13 ^c^	46.09 ± 3.92 ^c^	159.31 ± 5.49 ^a^	158.29 ± 9.19 ^a^
	Glutamic acid	23.17 ± 16.59 ^b^	6.13 ± 1.89 ^b^	2.30 ± 0.49 ^b^	28.01 ± 19.83 ^b^	811.48 ± 40.87 ^a^
Sweet amino acid	Theanine	37.63 ± 3.16 ^a^	2.79 ± 1.52 ^c^	21.68 ± 6.30 ^b^	34.25 ± 2.31 ^a^	10.31 ± 1.27 ^c^
	Asparagine	12.75 ± 4.59 ^d^	466.16 ± 34.85 ^b^	4.01 ± 0.29 ^d^	236.77 ± 68.77 ^c^	1406.01 ± 87.42 ^a^
	Glycine	134.05 ± 30.81 ^a^	146.09 ± 20.78 ^a^	76.5 ± 2.22 ^b^	27.36 ± 14.04 ^c^	13.14 ± 3.68 ^c^
	Alanine	116.81 ± 15.09 ^a^	37.97 ± 17.78 ^b^	31.85 ± 5.65 ^b^	44.09 ± 3.15 ^b^	93.19 ± 3.24 ^a^
	Serine	-	211.3 ± 23.67 ^a^	26.57 ± 6.00 ^b^	17.31 ± 0.52 ^b^	19.8 ± 6.80 ^b^
	Threonine	60.12 ± 23.46 ^ab^	35.84 ± 6.97 ^b^	35.37 ± 5.97 ^b^	59.18 ± 23.35 ^ab^	83.49 ± 7.40 ^a^
	Proline	88.06 ± 6.09 ^a^	44.54 ± 3.76 ^c^	64.27 ± 0.89 ^b^	29.06 ± 1.58 ^d^	11.45 ± 5.70 ^e^
Bitter amino acid	Histidine	34.5 ± 8.24 ^b^	-	-	110.88 ± 22.02 ^a^	155.38 ± 23.58 ^a^
	Arginine	70.33 ± 38.74 ^a^	-	-	183.42 ± 110.67 ^a^	199.58 ± 34.04 ^a^
	Valine	109.01 ± 21.08 ^d^	20.45 ± 8.25 ^c^	195.54 ± 4.79 ^a^	213.55 ± 4.16 ^a^	147.58 ± 9.18 ^b^
	Methionine	88.32 ± 40.00 ^a^	31.25 ± 5.69 ^b^	43.10 ± 2.71 ^ab^	90.45 ± 31.89 ^a^	90.90 ± 8.77 ^a^
	Isoleucine	103.68 ± 29.68 ^b^	71.28 ± 31.96 ^b^	249.78 ± 25.33 ^a^	126.97 ± 15.12 ^b^	227.02 ± 22.01 ^a^
	Leucine	32.44 ± 9.78 ^c^	21.66 ± 9.01 ^c^	73.81 ± 9.65 ^b^	24.78 ± 4.84 ^c^	171.22 ± 3.86 ^a^
	Aminobutyric acid	74.29 ± 32.23 ^b^	7.85 ± 4.06 ^c^	90.74 ± 3.34 ^b^	28.59 ± 6.19 ^c^	368.86 ± 5.81 ^a^
	Phenylalanine	48.34 ± 23.60 ^b^	15.58 ± 11.37 ^b^	-	59.44 ± 18.64 ^ab^	99.07 ± 3.31 ^a^
Odorless amino acid	Lysine	91.69 ± 30.7 ^a^	33.63 ± 5.22 ^b^	64.59 ± 4.26 ^ab^	45.54 ± 5.06 ^b^	88.71 ± 7.64 ^a^
	Cystine	3233.59 ± 844.51 ^a^	1352.87 ± 379.63 ^b^	3229.53 ± 204.24 ^a^	1505.03 ± 183.31 ^b^	1418.01 ± 71.89 ^b^
	Tyrosine	118.52 ± 9.52 ^a^	61.68 ± 18.31 ^c^	92.43 ± 2.78 ^b^	58.46 ± 7.12 ^c^	59.72 ± 5.09 ^c^
	Glutamine	195.09 ± 12.42 ^b^	-	265.65 ± 54.93 ^a^	-	55.82 ± 12.46 ^b^
Essential amino acid		533.6 ± 97.7 ^c^	229.69 ± 14.73 ^d^	662.2 ± 35.12 ^b^	619.92 ± 20.81 ^bc^	908.01 ± 43.7 ^a^
Non-essential amino acid		4153.81 ± 831.44 ^a^	2147.01 ± 398.82 ^b^	3812.62 ± 155.19 ^a^	2382.38 ± 140.27 ^b^	4382.07 ± 86.63 ^a^
Total amino acids		4799.34 ± 935.85 ^a^	2598.28 ± 186.24 ^b^	4604.63 ± 393.43 ^a^	3075.90 ± 140.8 ^b^	5689.03 ± 134.6 ^a^

“-” indicates that the device was not detected. The values of different lowercase letters in the same line differ significantly (*p* < 0.05).

**Table 3:** Substances with odor activity value (OAV) of volatile substances >1 in each variety.

Components	Odor Description ^a^	Odor Threshold ^b^ (mg/kg)	OAV				
			Zhongsang 5801	2000-3	Jialing-40	Yuesang10	White Shahtoot Mulberry
γ-Decanolactone	Fat, fruit, lactone, peach	0.0011 ^e^	- ^d^	-	-	-	709.09
γ-Nonlactone	Apricot, fruit	0.0021 ^e^	71.43	-	-	-	-
Butyrolactone	Caramel, cheese, roasted nut	>1 ^f^	-	-	-	>1.65	-
Ethyl decanoate	Brandy, grape, pear	0.005 ^g^	-	12	-	-	-
6-Methyl-2-heptanol	n.f ^c^	0.039∽0.40 ^h^	-	-	-	-	49.58~508.46
1-Dodecanol	n.f	0.016 ^i^	-	-	-	42.5	-
Hexaldehyde	Apple, fat, fresh, green, oil	0.005 ^g^	-	-	-	-	316
(E)-2-hexenal	n.f	0.0887 ^j^	-	-	-	-	130.78
2-Octenal	Dandelion, fat, fruit, grass, green, spice	0.003 ^k^	-	36.67	-	-	-
(Z)-4-decenaldehyde	Fruit	0.000004 ^l^	-	20,000	-	-	-
Nonanal	Fat, floral, green, lemon	0.0011 ^g^	127.27	163.64	100	1936.36	754.55
(E, Z)-2,6-nonadienal	Cucumber, green, wax	0.0008 ^g^	487.5	462.5	212.5	-	1837.5
2,4-Nonadienal	Cereal, deep fried, fat, watermelon, wet wool	0.0001 ^g^	-	1200	-	-	-
Decanal	Floral, fried, orange peel, penetrating, tallow	0.003 ^m^	-	-	-	-	30
2,6,6-Trimethyl-1-cyclohexene-1-carboxyaldehyde	n.f	0.003 ^n^	-	-	-	-	30
(E)-2-decenaldehyde	Fat, fish, orange	0.017~0.25 ^o^	-	-	-	-	0.5~5.88
2,4-Decadienal	Coriander, deep fried, fat, oil, oxidized	0.0003 ^p^	-	-	-	-	266.67
(2E, 4Z)-2,4-decadienal	n.f	0.00004 ^q^	-	-	-	-	1250
2-Octanone	Fat, fragrant, mold	0.0502 ^g^	409.36	213.35	198.21	1046.81	430.48
5-Hexyldihydro-2(3H)-furanone	n.f	0.0097 ^e^	-	24.74	-	-	-
maltol	n.f	1.24 ^r^	-	-	-	2.95	-
2,4-Di-tert-butylphenol	n.f	0.5 ^s^	1.92	2.46	3.7	23.74	2.22

^a^: Odor descriptors from https://www.femaflavor.org (accessed on 1 October 2024) ^b^: The odor threshold for each of the substances in aqueous medium, expressed in mg/kg. ^c^: n.f indicates an odor descriptor for an undetected substance. ^d^: “-” Indicates that the substance was detected in the undetected group and that the OAV value cannot be calculated. ^e^: Czerny et al. [46]. ^f^: Buttery et al. [47]. ^g^: Giri et al. [48]. ^h^: Schnabel et al. [49]. ^i^: Boonbumrung et al. [50]. ^j^: Burdack-Freitag et al. [51]. ^k^: Jorge et al. [52]. ^l^: Regina et al. [53]. ^m^: Melanie et al. [54,55]. ^n^: Rashash et al. [56]. ^o^: Tamura et al. [57,58]. ^p^: Kossa et al. [59]; Tressl et al. [60]. ^q^: Kerler et al. [61]; Kerscher et al. [62]. ^r^: Labbe et al. [63]. ^s^: Dietz et al. [64].

## Data Availability

The original contributions presented in this study are included in the article/Supplementary Materials. Further inquiries can be directed to the corresponding author.

## Supplementary Materials

The following supporting information can be downloaded at: www.mdpi.com/article/10.3390/foods13244088/s1, Table S1: Volatile components of five mulberry varieties (mg/kg).
