# Supplementary material for: Quality Characteristics and Flavor Analysis of Five Mulberry Varieties
Source: Foods. 2024 Dec 17;13(24):4088. doi: 10.3390/foods13244088 (PMC11675953; doi:10.3390/foods13244088)
Supplement: Supplementary file 1 [file foods-13-04088-s001.zip › foods-3310082-supplementary.pdf]

Supplementary Table S1. Volatile components of five mulberry varieties (mg/kg).

cultivar / volatile compounds		chemical formula	CAS	RT[1]	molar weig ht	RI[2]	Zhongsang 5801		2000-3		Jialing 40		Yuesang 10		White Shahtoot Mulberry	
							content	SI[3]	content	SI	content	SI	content	SI	content	SI
esters	phenyl isothiocyanate	C7H5NS	103-72-0	17.445	135	0	-	-	-	-	-	-	-	-	0.07±0.01a	95.00
	γ-decanolactone	C10H18O2	706-14-9	25.01833333	170	1383	-	-	-	-	-	-	-	-	0.78±0.17a	92.00
	γ-nonlactone	C11H20O2	104-67-6	22.1275	184	1483	0.15±0.04a	92.00	-	-	-	-	-	-	-	-
	diisobutyl phthalate	C16H22O4	84-69-5	34.4475	278	1908	0.15±0.11a	95.00	-	-	-	-	-	-	0.14±0.01a	94.00
	methyl acetate	C3H6O2	79-20-9	4.268333333	74	487	-	-	-	-	-	-	1.2±0.16a	91.00	-	-
	ethyl propyl oxalate	C7H12O4	0-00-0	5.7575	160	1052	-	-	-	-	-	-	0.81±0.06a	80.50	-	-
	butyrolactone	C4H6O2	96-48-0	8.71	86	825	-	-	-	-	-	-	1.65±1.1 1a	90.00	-	-

	bis (2-methylpropyl) 1,2-phthalate	C16H22O4	84-69-5	35.3475	278	1908	-	-	-	-	-	-	2.56±2.35a	94.00	-	-
	dibutyl phthalate	C16H22O4	84-74-2	36.25	278	2037	0.05±0.04a	92.00	-	-	-	-	1.01±0.85a	94.67	1.01±0.85a	96.00
	L- homoserine lactone	C6H9NO3	51524-71-1	15.40166667	143	386	-	-	-	-	-	-	-	-	0.14±0.04a	84.50
	ethyl decanoate	C12H24O2	110-38-3	23.09	200	1381	-	-	0.06±0.00a	92.67	-	-	-	-	-	-
	ethyl dodecanoate	C14H28O2	106-33-2	28.24166667	228	1580	-	-	0.08±0.01a	92.67	-	-	-	-	-	-
	ethyl hexadecanoate	C18H36O2	628-97-7	36.89	284	1978	-	-	0.08±0.01a	95.00	0.05±0.01a	93.00	1.09±0.35a	93.00	-	-
	methyl 9,12-octadecadienoate	C19H34O2	2462-85-3	38.38	294	2093	-	-	0.06±0.01a	94.67	-	-	-	-	-	-
alcohols	(R,R)-2,3-Butanediol	C4H10O2	24347-58-8	5.665	90	743	-	-	0.05±0.00a	95.50	-	-	-	-	-	-
	2- furfuryl methanol	C5H6O2	98-00-0	7.25	98	885	-	-	0.1±0.05a	91.00	-	-	-	-	-	-
	2-ethyl hexanol	C8H18O	104-76-7	12.235	130	995	0.08±0.06a	95.00	-	-	-	-	0.19±0.03a	93.00	-	-
	6- methyl -2-heptanol	C8H18O	4730-22-7	11.66666667	130	915	-	-	-	-	-	-	-	-	19.83±8.63a	92.67

	α-butyl -1-cyclopropyl -1-pentanol	C8H16O	4379-16-2	14.25333333	128	981	-	-	-	-	-	-	-	-	1.08±0.12a	88.33
	1- dodecanol	C12H26O	112-53-8	25.23	186	1457	-	-	-	-	-	-	0.68±0.14a	94.00	-	-
	pentadecanol	C15H32O	629-76-5	34.905	228	1755	-	-	-	-	-	-	1.21±0.73a	96.00	-	-
	nonadecanol	C19H40O	454-84-8	38.2625	284	2153	-	-	-	-	-	-	0.67±0.27a	95.00	-	-
aldehydes	hydroxyacetaldehyde	C2H4O2	141-46-8	3.469	60	651	-	-	0.36±0.10a	94.33	-	-	1.38±0.85a	95.50	0.18±0.04a	96.50
	hexaldehyde	C6H12O	66-25-1	5.0325	100	806	-	-	-	-	-	-	-	-	1.58±0.24a	93.00
	(E)-2- hexenal	C6H10O	6728-26-3	6.485	98	814	-	-	-	-	-	-	-	-	11.6±2.00a	95.00
	2- octenal	C8H14O	2548-87-0	13.02	126	1013	-	-	0.11±0.02a	94.50	-	-	-	-	-	-
	glutaric dialdehyde	C5H8O2	111-30-8	14.175	100	895	-	-	0.25±0.08a	84.50	-	-	-	-	-	-
	(Z)-4-decenaldehyde	C10H18O	21662-09-9	14.27	154	1212	-	-	0.08±0.01a	89.50	-	-	-	-	-	-
	nonanal	C9H18O	124-19-6	14.540625	142	1104	0.14±0.06a	96.00	0.18±0.02a	94.33	0.11±0.01a	93.67	2.13±1.51a	92.50	0.83±0.15a	91.00
	(E, Z)-2,6-nonadienal	C9H14O	557-48-2	16.037	138	1120	0.39±0.1b	94.00	0.37±0.02b	92.67	0.17±0.04b	94.33	-		1.47±0.16a	92.67

	(E)-2- nonenal	C9H16O	0-00-0	16.28111111	140	1112	1.56±0.46b	96.00	1.99±0.08b	89.33	0.51±0.20c	96.00	3.07±0.38a	96.50	1.39±0.05b	96.00
	2,4-nonadienal	C9H14O	5910-87-2	17.9525	138	1120	-	-	0.12±0.01a	87.50	-	-	-	-	-	-
	(Z)-9- hexadecenal	C16H30O	56219-04-6	39.10333333	238	1808	-	-	-	-	-	-	2.81±1.61a	90.00	-	-
	decanal	C10H20O	112-31-2	17.7	156	1204	-	-	-	-	-	-	-	-	0.09±0.03a	94.00
	2,6,6- trimethyl -1-cyclohexene -1-carboxyaldehyde	C10H16O	432-25-7	18.085	152	1204	-	-	-	-	-	-	-	-	0.09±0.01a	89.50
	5-hydroxymethylfurfural	C6H6O3	67-47-0	18.339	126	1163	-	-	-	-	-	-	11.38±3.78a	88.33	0.42±0.12a	92.00
	(E)-2-decenaldehyde	C10H18O	3913-81-3	19.365	154	1212	-	-	-	-	-	-	-	-	0.1±0.03a	91.50
	2,4- decadienal	C10H16O	2363-88-4	20.31333333	152	1220	-	-	-	-	-	-	-	-	0.08±0.00a	93.00
	(2E, 4Z)-2,4-decadienal	C10H16O	25152-83-4	20.99666667	152	1220	-	-	-	-	-	-	-	-	0.05±0.01a	91.67
	2- undecenal	C11H20O	2463-77-6	19.365	168	1311	-	-	-	-	-	-	-	-	0.07±0.00a	90.50
ketones	hydroxypropanone	C3H6O2	116-09-6	3.956875	74	698	-	-	0.14±0.10a	96.00	0.1±0.03a	96.00	4.72±3.7a	95.33	0.26±0.01a	97.00
	2(5H)- furanone	C4H4O2	497-23-4	8.65	84	807	-	-	-	-	-	-	0.21±0.01a	92.50	-	-

	6- oxabicyclo [3. 1. 0] hexan -3- one	C5H6O2	74017-10-0	8.9275	98	782	-	-	-	-	-	-	7.4±5.87a	87.00	-	-
	dihydroxy acetone	C3H6O3	96-26-4	9.35	90	941	-	-	-	-	-	-	5.73±1.91a	90.00	0.63±0.1a	91.00
	2-octanone	C8H16O	111-13-7	10.8133	128	952	20.55±8.31bc	96.50	10.71±1.27bc	98.00	9.95±1.29c	97.67	52.55±4.21a	95.50	21.61±2.69b	96.00
	2,3- dihydro -3,5- dihydroxy -6- methyl -4(H)- pyran -4- one	C6H8O4	28564-83-2	15.74166667	144	1269	-	-	0.28±0.21a	93.50	-	-	8.81±2.39a	94.00	-	-
	5-hexyldihydro-2(3H)-furanone	C9H16O2	104-61-0	22.115	156	1284	-	-	0.24±0.01a	96.67	-	-	-	-	-	-
acids	formic acid	CH2O2	64-18-6	3.405833333	46	0	-	-	0.68±0.38b	96.50	-	-	14.87±12.35^{a}	94.50	0.43±0.06b	94.50
	methyl tartaric acid	C4H6O5	595-98-2	9.92	134	1223	-	-	-	-	-	-	-	-	1.17±0.19	95.50
	enanthic acid	C7H14O2	111-14-8	13.78	130	1073	-	-	-	-	-	-	-	-	0.1±0.01	86.50
	lauric acid	C12H24O2	143-07-7	27.40833333	200	1570	-	-	0.11±0.01a	94.00	-	-	0.54±0.31a	94.00	-	-
	tetradecanoic acid	C14H28O2	544-63-8	32.118	228	1769	-	-	0.1±0.02a	94.00	-	-	1.03±0.52a	94.00	-	-
	pentadecanoic acid	C15H30O2	1002-84-2	36.3425	242	1869	-	-	0.35±0.16b	91.00	0.27±0.13b	92.00	7.36±2.91a	92.33	0.33±0.06b	92.67

	10(E), 12(Z)-conjugated linoleic acid	C18H32O2	2420-56-6	38.9975	280	2183	-	-	-	-	-	-	3.69±1.73a	95.50	-	-
	(Z)-6- octadecenoic acid	C18H34O2	593-39-5	39.1125	282	2175	-	-	-	-	-	-	1.02±0.52a	90.00	-	-
	linoleic acid	C18H32O2	544-71-8	39.24	280	2183	-	-	-		0.08±0.02a	92.00	-	-	-	-
other	ammonium acetate	C2H7NO2	631-61-8	3.625909091	77	630	0.16±0.01b	96.00	1.45±0.31b	92.67	0.42±0.35b	90.00	4.61±1.52a	96.00	0.9±0.11b	97.00
	butadiene dioxides	C4H6O2	1464-53-5	5.11	86	600	-		-		0.03±0.00a	86.50	-	-	0.14±0.00a	86.67
	oxime methoxyphenyl	C8H9NO2	0-00-0	9.24	151	1301	0.78±0.73a	84.50	0.32±0.07a	86.33	0.53±0.13a	84.00	-	-	1.06±0.3a	83.67
	1-butylcyclopentene	C9H16	2423-01-0	12.21	124	969	-	-	-	-	-	-	-	-	1.32±0.45a	86.33
	2,3-dihydro-3,5-dihydroxy -6-methyl	C6H8O4	28564-83-2	15.81833333	144	1269	-	-	-	-	-	-	-	-	2.26±0.4a	93.00
	octyl cyclopropane	C11H22	1472-09-9	18.9	154	1117	-	-	-	-	-	-	-	-	0.19±0.02a	84.00
	8- methyl -1-undecene	C12H24	74630-40-3	21.5525	168	1140	-	-	-	-	-	-	-	-	0.05±0.01a	82.00
	maltol	C6H6O3	118-71-8	11.38	126	1063	-	-	-	-	-	-	3.66±3.50a	91.00	-	-

2,4- di-tert-butylphenol	C14H22O	96-76-4	26.04666667	206	1555	0.96±0.08b	95.33	1.23±0.37b	96.00	1.85±0.22b	95.00	11.87±2.14a	94.33	1.11±0.33b	96.00
tetradecane	C14H30	629-59-4	25.835	198	1413	-	-	-	-	-	-	0.23±0.19a	94.50	-	-

[1]RT: retention time. [2]RI: retention index. [3]SI: Similarity index. The values of different lowercase letters in the same line differ significantly ($P < 0.05$) ; “-” Indicates that the device is not detected. Retention time and SI represent averages.
